# MAXIMIZING THE SCALABILITY OF THE CHRONIC DISEASE SELF-MANAGEMENT PROGRAM AMONG OLDER ADULTS IN STATE PRISONS

**DOI:** 10.1093/geroni/igae098.0525

**Published:** 2024-12-31

**Authors:** Rodlescia Sneed

**Affiliations:** Wayne State University, Detroit, Michigan, United States

## Abstract

Incarcerated older adults have high rates of chronic disease; however, there is limited chronic disease programming in most state prison systems. The Chronic Disease Self-Management Program (CDSMP) is an evidence-based program that has been used within state prisons on a limited basis, but there has been little research into efficient and effective strategies for scaling up the intervention within state prisons. Scale-up (i.e. deliberate efforts to increase the impact of successfully tested health innovations to benefit more people and promote sustainability) is an understudied concept with few existing empirical studies explicitly focused on the scale-up process. Assessing scalability, however, is crucial for ensuring sustainability of complex interventions within resource-poor settings. The purpose of this study was to evaluate and maximize the scalability of CDSMP among older adults in state prisons. Guided by the Scaling up Management Framework, we conducted interviews with representatives from 10 U.S. state prison systems on ways to maximize the scalability of CDSMP within state correctional settings. Participants provided information in numerous areas related to scale-up, including strategies for achieving buy-in, special situations where implementation might be especially difficult, potential funding strategies, and ideal partners for scale-up efforts. As the prison population continues to age, the burden of chronic disease within correctional systems will continue to increase, which contributes to skyrocketing correctional costs. Understanding how to expand evidence-based chronic disease programs within correctional systems is crucial for reducing disease-related morbidity and mortality among incarcerated individuals and for reducing costs.

